# Expanding the Knowledge on the Skillful Yeast *Cyberlindnera jadinii*

**DOI:** 10.3390/jof7010036

**Published:** 2021-01-09

**Authors:** Maria Sousa-Silva, Daniel Vieira, Pedro Soares, Margarida Casal, Isabel Soares-Silva

**Affiliations:** 1 Centre of Molecular and Environmental Biology (CBMA), Department of Biology, University of Minho, Campus de Gualtar, 4710-057 Braga, Portugal; m.silva@bio.uminho.pt (M.S.-S.); jdanav@gmail.com (D.V.); pedrosoares@bio.uminho.pt (P.S.); mcasal@bio.uminho.pt (M.C.); 2 Institute of Science and Innovation for Bio-Sustainability (IB-S), University of Minho, 4710-057 Braga, Portugal

**Keywords:** *Cyberlindnera jadinii*, phylogeny, life cycle, genome, physiology, biotechnology applications, membrane transporter systems

## Abstract

*Cyberlindnera jadinii* is widely used as a source of single-cell protein and is known for its ability to synthesize a great variety of valuable compounds for the food and pharmaceutical industries. Its capacity to produce compounds such as food additives, supplements, and organic acids, among other fine chemicals, has turned it into an attractive microorganism in the biotechnology field. In this review, we performed a robust phylogenetic analysis using the core proteome of *C. jadinii* and other fungal species, from Asco- to Basidiomycota, to elucidate the evolutionary roots of this species. In addition, we report the evolution of this species nomenclature over-time and the existence of a teleomorph (*C. jadinii*) and anamorph state (*Candida utilis*) and summarize the current nomenclature of most common strains. Finally, we highlight relevant traits of its physiology, the solute membrane transporters so far characterized, as well as the molecular tools currently available for its genomic manipulation. The emerging applications of this yeast reinforce its potential in the white biotechnology sector. Nonetheless, it is necessary to expand the knowledge on its metabolism, regulatory networks, and transport mechanisms, as well as to develop more robust genetic manipulation systems and synthetic biology tools to promote the full exploitation of *C. jadinii*.

## 1. Introduction

The future of our society challenges researchers to find novel technologies to address global environmental problems, mitigate ecosystems’ damage, and biodiversity losses, as the current model of development based on natural resources exploitation is unsustainable. Exploring microorganisms for the production of platform chemicals constitutes an alternative approach to avoid the use of nonrenewable petrochemical-based derivatives. Developing applications for the industrial sphere using biological systems instead of classical chemical catalysts is the main focus of white biotechnology [1]. In microbial-based industrial processes, several features have to be addressed to obtain robust cell factories capable of achieving superior metabolic performances, such as the optimization of metabolic fluxes, membrane, and transporter engineering, and increased tolerance against harsh industrial conditions and toxic compounds [2]. In addition, specifications like the cost of feedstock, product yield and productivity, and downstream processing have to be taken in account to develop successful industrial approaches [1]. Withal the fact that *Saccharomyces cerevisiae* is by far the most relevant industrial yeast species, *Cyberlindnera jadinii* is an example of the so-called non-*Saccharomyces* yeasts [3] claiming for a place as a relevant contributor to the industrial biotechnological sector. The yeast *C. jadinii* is able to produce valuable bioproducts being an attractive source of biomass enriched in protein and vitamins. The richness of protein content, around 50% of dry cell weight, and amino acid diversity turn its biomass ideal as a source of protein supplement for animal feed and human consumption [4]. The high degree of tolerance to environmental changes occurring during fermentation turn *C. jadinii* an alternative to other established cell factory systems [5]. As a Crabtree-negative yeast, it has one of the highest respiratory capacities among characterized yeast species, being considered ideal for continuous cell cultures [6]. The Food and Drug Administration (FDA) attributed the “General Regarded as Safe” (GRAS) status to this yeast, recognizing it as safe and suitable for supplying food additives and dietary supplements for humans [5,7,8,9]. The ability to produce relevant compounds, to grow in a wide range of temperatures, to use inexpensive media with high productivity levels turns it an industrially relevant microorganism [8,10,11,12]. Recent efforts have developed *C. jadinii* molecular tools for metabolic engineering processes and the overexpression of proteins. In the past, the uncertainty of this yeast polyploidy, together with the lack of suitable selection markers and expression cassettes, [10] delayed its widely use as cell factory. With this review, we intend to compile the existing knowledge on this yeast, that will allow the development of future strategies to strengthen the role of *C. jadinii* in the biotechnology sector. We start by reviewing the nomenclature of this species, altered several times over time. An update on the current nomenclature of the most relevant strains is also presented. We establish the evolutionary relationship of *C. jadinii* within other fungi with a complete genome available. The most relevant morphological and physiological traits are also here described, together with the genetic manipulation tools and expression systems currently available. Moreover, we present a summary of all the plasma membrane transporter systems so far characterized in this yeast, as they are key-players for cell factory optimization. Finally, we will focus on the biotechnological potential of this yeast and highlight the future challenges to achieve the full exploitation of this industrially valuable microorganism.

## 2. Ecology, Taxonomy, and Evolution

The natural environment of *Cyberlindnera jadinii* is still an open question. It is thought that it may be associated with the decomposition of plant material in nature, as it is able to assimilate pentoses and tolerate lignocellulosic by-products [3], displays great fermentative ability, and is copiotrophic [13]. The current laboratory strains were isolated from distinct environments, namely, from the pus of a woman abscess (CBS 1600/NRRL Y-1542), a cow with mastitis (CBS 4885/NRRL Y-6756), a yeast deposit from a distillery (CBS 567), yeast cell factories (CBS 621), and flowers (CBS 2160) [5,6,7,8,9,12]. The extensive nomenclature revisions of this species are well described in “The tortuous history of *Candida utilis*” by Barnet [14]. In 1926, this yeast was isolated from several German yeast factories, which had been cultivated without a systematic name during the time of World War I for food and fodder [14]. It was first named *Torula utilis* being later referred to as *Torulopsis utilis* (1934). The “food yeast” was also designated as *Saccharomyces jadinii* (1932), *Hansenula jadinii* (1951), *Candida utilis* (1952), *Pichia jadinii* (1984), and *Lindnera jadinii* (2008) [12,14,15,16]. From the aforementioned, *C. utilis* was the nomenclature most commonly used, having almost 1000 published papers in PubMed (results available at: https://pubmed.ncbi.nlm.nih.gov/?term=%22candida+utilis%22; Accessed 16 October 2020). The *Candida* genus comprised species that form pseudohyphae or true hyphae with blastoconidia, among other standard characteristics [17,18,19]. In the classification system implemented in 1952 by Lodder and Kreger-van Rij, the *Candida* genus included yeasts that produce only simple pseudohyphae [14]. At that time, the majority of the isolates was renamed as *C. utilis* [18]. Later, the *C. utilis* was established as an asexual state of a known ascosporogenous yeast, *Hansenula jadinii*, as it was found to share some similarities between phenotypic traits [20]. In 1984, even though concurring with a publication of an extensive chapter of the genus *Hansenula*, Kurtzman moved most of the *Hansenula* species to the *Pichia* genus, due to their “deoxyribonucleic acid relatedness.” Thus, *C. utilis* was renamed *Pichia jadinii*. A quarter of a century later, this yeast species was again renamed as *Lindnera jadinii* based on analyses of nucleotide sequence divergence in the genes coding for large and small-subunit rRNAs [12]. Species integrated into the *Lindnera* differ considerably in ascospore morphology ranging from spherical to hat-shaped or Saturn-shaped spores. In addition, this clade includes both hetero- and homothallic species and physiological features as fermenting glucose and assimilating a variety of sugars, polyols, and other carbon sources are defining characteristics of the *Lindnera* genus [12]. Finally, 1 year later, the genus *Lindnera* was replaced by *Cyberlindnera*, as the later homonym defined a validly published plant genus [15]. This substitution occurred in 21 new species, including *Cyberlindnera jadinii* [15]. In summary, any of the aforementioned nomenclatures reported in the literature may refer to the same organism since *C. utilis* is the anamorph state and *C. jadinii* the teleomorph state [14]. The anamorph represents the asexual stage of a fungus contrasting with the teleomorph form that defines the sexual stage of the same fungus [21]. The primary name of a species relies on the sexual state or teleomorph, but a second valid name may rely on the asexual state or anamorph [22]. However, this should only happen when teleomorphs have not been found for a specific species or it is not clear if a particular teleomorph is the same species as a particular anamorph. Accordingly, since 2013, the International Botanical Congress states that the system for allowing separate names for the anamorph state should end [23]. The new International Code of Nomenclature for algae, fungi, and plants, the Melbourne Code, supports the directive that fungal species or higher taxon should be assigned with a single valid name. Accordingly, anamorph yeast genera like *Candida* should be revised to turn the genus consistent with phylogenetic affinities [19]. Notwithstanding, the reclassification of several *C. utilis* as *C. jadinii* in several culture type collections is still confusing, reaching a point where the same strain is designated as *C. utilis* and *C jadinii* in different culture type collections. This aspect, together with the previous nomenclatures used in research papers, leads to unnecessary misunderstandings. To clarify this, Table 1 presents the alternative designations of the main laboratory strains.

**Figure 1:**
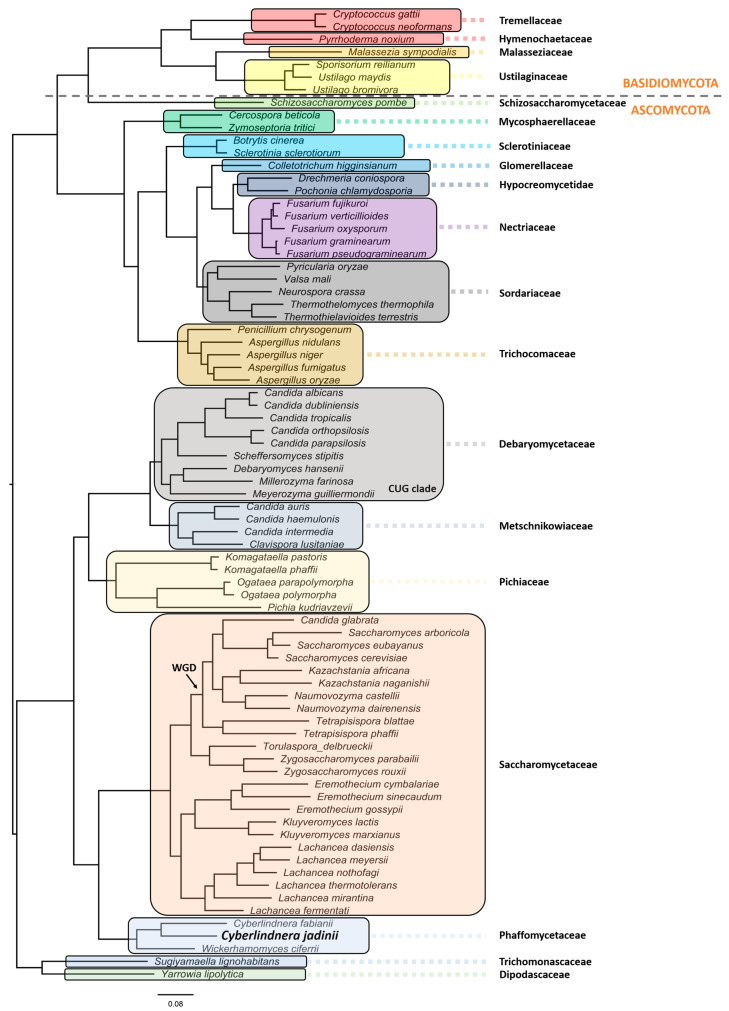
Evolutionary relationship of *Cyberlindnera jadinii*, a member of the Phaffomycetaceae clade. The phylogenetic reconstruction was obtained using the following parameters: maximum likelihood in IQ-TREE (http://www.iqtree.org), the model of amino acid evolution JTT (Jones-Taylor-Thornton), and four gamma-distributed rates. Homologues were detected for 1567 proteins across the proteome of 77 selected fungal species from NCBI. The 1567 set of proteins were aligned and then concatenated in order to use in the phylogenetic analysis. These proteins offer a clear high-resolution evolutionary view of the different species, as they are essential proteins beyond the specific biology of the different yeasts. Bootstrapping provided values of 100% for all the nodes. Yeast and fungi families are highlighted with different colors and shades. The phylogenetic relationships reflect evolutionary ancestries, independently of adaptations and overall gene contents within the various species. All families with more than one representative species in the analyses formed monophyletic groups.

*C. jadinii* belongs to the phylum Ascomycota, subphylum Saccharomycotina. The members of this subphylum constitute a monophyletic group of ascomycetes that are well defined by ultrastructural and DNA characteristics [13]. These include lower amounts of chitin in overall polysaccharide composition at cell walls, being unable to stain with diazonium blue, low content of guanine and cytosine (G + C < 50%) at nuclear DNA, and presence of continuous holoblastic bud formation with wall layers. *C. jadinii* belongs to the Saccharomycetes class, Saccharomycetidae family, Saccharomycetales order, and the *Cyberlindnera* genus. However, a comprehensive phylogenetic analysis and evolutionary relationship are still missing for this species [36,37,38]. Aiming at filling this gap, we performed a robust phylogeny reconstruction [36,39,40,41]. As can be depicted in Figure 1, this yeast localizes in the Phaffomycetaceae clade together with *Cyberlindnera fabianii* and *Wickerhamomyces ciferri*. The nearest neighbors belong to the Saccharomycetaceae family, which includes a clade with *S. cerevisiae/Torulaspora delbrueckii* species and another clade with *Eremothecium gossypii* (former *Ashbya gossypii*), *Kluyveromyces lactis/marxianus*, and *Lachancea* species. Despite the previous genus nomenclature adopted for *C. jadinii* (Candida and Pichia), it is phylogenetically distant from the Debaryomycetaceae and Pichiaceae families that include the *Candida* species, except *C. glabrata*, the *Pichia kudriavzevii*, and *Ogataea* species. *Komagataella phaffi* is as expected included in the Pichiaceae clade, together with *Komagataella pastoris* [36,40]. The Trichomonascaceae/ Dipodascaceae clade, formerly known as the *Yarrowia* clade, includes now the *Sugiyamaella lignohabitans* species together with *Yarrowia lipolytica*, and is the most distant yeast clade, except for the Schizosaccharomycetaceae that clusters with all the Basiodiomycota. The filamentous fungi *Neurospora crassa* and *Fusarium graminearum* are in different clades as members of the Sordariaceae and Nectriaceae clades, respectively [40]. In addition, in the Sordariaceae clade, the phylogenetic position of *Thermothelomyces thermophila* species (*Myceliophthora thermophila*) was uncovered [39,42].

## 3. Life Cycle and Genome Organization

Kurtzman and colleagues proposed *C. jadinii* as the teleomorphic parental species of *C. utilis*, due to the 85% reassociation rate obtained between genomic DNA of the two yeast species and to the high similarities of ribosomal RNA sequences [20,34]. The formation of ascospores allied with genomic sequencing data confirmed the diploidy of *C. jadinii* NRRL Y-1542 strain and the identification of *MAT*a and *MAT*α genes allelic locations [20,37]. Ikushima et al. studied the polyploid of several *C. utilis* strains (NBRC0396, 0619, 0626, 0639, 0988, 1086, and 10707) detecting an overall ploidy switching between 2n and 5n [43]. Later, Kondo and colleagues inferred, through the analysis of *C. utilis* ATCC 9950 transformants the presence of a diploid state, although some years later, the sequential disruption of the *URA3* and *PDC1* locus suggested the tetraploidy of this strain [25,43,44]. A fluorescence-activated cell sorting analysis pointed out a ploidy of 3n to 5n in this latter *C. utilis* strain, following the aforementioned data by Ikushima et al. [16,43]. Furthermore, a single nucleotide polymorphism analysis suggested that the *C. utilis* NBRC0988 genome was triploid [16,25,44]. Overall, these results suggested that *C. utilis* has derived from the parental yeast *C. jadinii* through triploidization pursuing an unexplained sequence of genetic events [16].

Recently, Krahulec and colleagues determined the *C. utilis* CCY 39-38-18 genome ploidy through the analysis of the copy number of the maltase gene in deleted mutants, pointing out the tetraploidy of this strain [32]. Despite the existing ploidy variation, the diploid state of *C. jadinii* impelled its genetic manipulation and subsequent utilization in the biotechnological industry [16,37]. Table 2 summarizes the genetic features of the *C. jadinii* strains sequenced so far [24]. Although the GC-content is quite similar, the genome size is different among the distinct strains evaluated (Table 2). When compared to *S. cerevisiae*, *C. jadinii* has an increased genome size and higher GC-content. The genomic features of *C. jadinii* strains are different from two closed phylogenetic species *Wickerhamomyces ciferrii and* *Cyberlindnera fabianii* (Figure 1) that, respectively, contain a G-C ratio of 30.4% and 44.4%, genome sizes of 15.9 and 12.3 Mb, and a total of 6702 and 5944 CDS [45,46].

Among the genomic indicators presented here, the genome size and predicted/protein-coding genes seem to be strain dependent, whereas the GC-content is species independent, which is in accordance with the previously reported ploidy variations. Some differences were also detected among *C. jadinii* strains considering their specific genetic features, namely, the NBRC 0988 strain has 6417 predicted open reading frames (ORFs) comprising 16 unique ORFs [5,10,24], whereas the CBS1600 strain has 5689 ORFs, including 64 unique ORFs [10]. In 2015, Rupp and colleagues revealed a close haploid consensus sequences sizes, 12.7 Mbp for *C. jadinii* and 12.8 Mbp for *C. utilis* with an overall sequence identity of 98% [16].

## 4. Morphology and Physiology

The *C. jadinii* microscopic view provided by Kurtzman et al. (2011) has shown the diversity of cell shapes and sizes [20,34] after 10–30 days at 25 °C in 5% Malt Extract Agar media. The cell patterns of *C. jadinii* CBS 1600 varied from ellipsoidal to elongated occurring in single cells or in pairs. Some pseudohyphae forms were also detected with diameter balanced between (2.5–8.0) and (4.1–11.2) µm.

**Figure 2:**
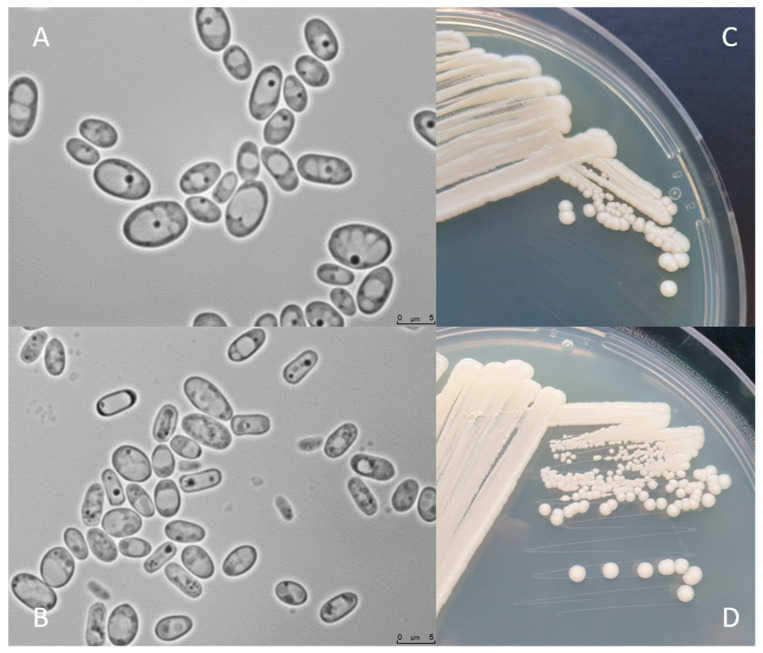
*C. jadinii* DSM 2361 morphological traits. (**A**) Microscopic photographs of *C. jadinii* cells after 3 days (**A**) and 12 days (**B**) of growth at 30 °C on yeast extract-peptone-dextrose media. Scale bars are 5.0 µm. Macro-morphological features of *C. jadinii* after 3 days of growth in YPD (**C**) and Malt Extract Agar (**D**) media, at 30 °C.

Figure 2 shows *C. jadinii* DSM 2361 strain cultivated on YPD or Malt Extract Agar media for 3 (A and C) and 12 days (B and D) at 30 °C. The colonies are white, round, with a smooth texture, an entire margin, and a convex elevation trait (C and D). Cells present an ellipsoidal to elongated form, with a diameter between 5 and 7.5 µm (A and B). Yeast cell morphology can be tightly influenced by the environment. These modifications can affect the fermentation performance by inducing rheological changes that can influence mass and heat transfer alterations in the bioreactor [49]. However, in a study performed by Pinheiro et al. (2014), the CBS 621 strain cultured in a pressurized-environment triggered with 12 bar air pressure presented no significant differences in cell size and shape [35]. *C. jadinii* is a homothallic species and forming hat-shaped ascospores that can be present in a number of one to four in unconjugated deliquescent asci [34]. *Cyberlindnera* species can assimilate several compounds, namely, sugars and organic acids. The robust fermentation characteristics of *C. jadinii* allow growth in a diversity of substrates from biomass-derived wastes, including hardwood hydrolysates from the pulp industry, being able to assimilate glucose, arabinose, sucrose, raffinose, and D-xylose [8,9,10,50]. As previously mentioned, *C. jadinii* is a Crabtree-negative yeast, reaching higher cell yields under aerobic conditions [51,52,53] than Crabtree-positive species. The Crabtree-negative effect favors the respiration process over fermentation, enabling the development of phenotypes relevant for protein production [54]. This species has a high tolerance to elevated temperatures, being able to grow in a broad spectrum of temperatures from 19 to 37 °C [37] and to tolerate long-term mild acid pHs (~3.5) [55]. Another relevant property is the ability to release proteins to the extracellular medium [56]. Significant lipase and protease content were achieved using a wild *C. jadinii* strain isolated from spoiled soybean oil, using solid-state fermentation [57,58]. *C. jadinii* assimilates alcohols, acetaldehyde, organic acids, namely, monocarboxylates (DL-lactate), dicarboxylates (succinate), and tricarboxylates (citrate), sugar acids (D-gluconate), and various nitrogen sources comprising nitrate, ammonium hydroxide, as well as amino acids [8,10,12,37]. A set of metabolic advantages, as the high metabolic flux in TCA cycle, the great amino acid synthesis ability, and strong protein secretion turns *C. jadinii* a yet underexplored host for bioprocesses. An incomplete understanding of genetics, metabolism, and cellular physiology combined with a lack of advanced molecular tools for genome edition and metabolic engineering manipulation of *C. jadinii* hampered its development for cell factory utilization.

## 5. Molecular Tools for *C. jadinii* Manipulation

The genetic manipulation of *C. jadinii* has enabled the heterologous expression of various genes, resulting in the improvement of metabolic traits targeted for the optimization of exogenous product formation [8,10,44,56]. The establishment of genetic transformation methods allowed the efficient production of enzymes, carotenoids compounds, and organic acids such as L-lactic acid [44,56,59,60]. The development of an integrative transformation vector for the *C. jadinii* ATCC 9226 and *C. utilis* ATCC 9256 strains relied on a gene encoding a mutated ribosomal protein L41, conferring cycloheximide resistance as a dominant selection marker [25]. Dominant markers were used for cell transformation of *aph*, *hph*, *nat*, and *ble* genes, conferring resistance to G418, hygromycin B, nourseothricin, or zeocin, respectively, and the endogenous gene *YAP1*, conferring cycloheximide resistance [56,60,61,62]. A multiple gene disruption method based on the Cre-loxP system allowing the reuse of selection markers was developed for *C. jadinii* NBRC0988 [43]. Auxotrophic *ura3* strains were transformed with the expression vectors further integrated in the rDNA locus or in other chromosomal loci (e.g., *TDH3*). Single-plasmid integrations were reported for the *TDH3* and *HIS3* loci [16,25,56] as well as multiple plasmid integrations for the rDNA and *URA3* loci for the expression of heterologous genes in high copy number (up to 90 copies) [63]. High plasmid stability was observed mainly for integrations into the *URA3* and *HIS3* loci, in contrast to the integrations in the rDNA, while integrations at the *TDH3* locus were reported to be both stable and unstable [56,64,65]. Two chromosomal autonomously replicating sequences (ARS) were uncovered in *C. jadinii* ATCC 9226 and *C. utilis* ATCC 9256. Six plasmids harboring these ARS were obtained using a G418-resistance marker. The low copy number plasmids pCARS6 (CuARS1 region) and pCARS7 (CuARS2 region) presented the highest transformation efficiency [26]. A set of promoters were also explored to promote an efficient expression in *C. jadinii*, the *TDH3*, homolog of *TDH3* from *S. cerevisiae*, encoding glycerol-3-phosphate dehydrogenase, as well *PGK1* and *PDC1* promoters from *C. jadinii* NBRC0988 encoding the phosphoglycerate kinase and pyruvate decarboxylase, respectively [44,64]. Furthermore, Kunigo et al. identified the highly xylose-inducible, glucose-repressed promoters of *XDH1* and *GXS1* genes, encoding a NAD-xylitol dehydrogenase and glucose/xylose symporter, respectively [56]. Promoters of the genes encoding the plasma membrane ATPase Pma1, Rpl29/Rpl31 ribosomal proteins, and Rpl41, as well as P14/P57 promoters from unknown chromosomal loci were used for the production of valuable products for the food industry, namely, for the secretion of heterologous proteins [25,66,67,68]. CRISPR-Cas9 has quickly become the preferred targeted genome-editing technology for the genetic manipulation of yeasts [69], being extensively used in *S. cerevisiae*. The adaptation of the type II CRISPR/Cas system has been successfully used for the genetic manipulation of non-*Saccharomyces* species, such as *Y. lipolytica*, *K. pastoris*, *K. lactis*, *Schizosaccharomyces pombe*, and some pathogenic yeast species, such as *Candida albicans* and *Cryptococcus neoformans* [70,71]. Recently, a patent application reported the development of a CRISPR/Cas9 system that was applied to knock out and insert exogenous genes in *C. jadinii* ATCC 22023 [72]. This strategy also uncovered the triploidy of this strain.

## 6. Emerging Biotechnological Applications

### 6.1. Therapeutic Applications

Being an edible yeast, *C. jadinii* has the potential to target the gastrointestinal tract in humans and animals [10,55]. *C. jadinii*’s robust growth characteristics including insensitivity to low pH and temperatures up to 40 °C, allow its transit in the gastrointestinal tract without losing viability [55,73]. Additionally, like other food constituents, intact/partially degraded *C. jadinii* cells may adhere to M cells in the small intestine. Upon this, they are translocated to antigen-presenting cells of Peyer’s plaques or to other lymphoid tissue connected with the gastrointestinal tract [74,75]. The ingestion of engineered *C. jadinii* cells, carrying a myelin oligodendrocyte glycoprotein antigen on its surface, promoted tolerance to self-antigens in a mouse model of the autoimmune multiple sclerosis (MS) disease [76]. *C. jadinii* cells expressing the immunodominant MOG_35–55_ epitope of a myelin protein on their surface, fused with the native fungal Gas1 cell wall protein, prevented the typical MS symptoms in this animal model [76]. The cell surface display of antigens by *C. jadinii* seems to modulate immune responses, either by suppression to combat autoimmune disease or through immune stimulation, enabling the creation of edible vaccines [73,76,77]. *C. jadinii* cells were also applied as probiotic agents against fungal infections (particularly oral candidiasis) as an antagonist to the relevant human fungal pathogens *Candida albicans* (strains SC5312, 10341, and GDH2346), *Aspergillus* sp., and *Fusarium* sp. [73]. The unidentified toxins secreted by *C. jadinii* act as antagonistic compounds conditioning the growth, systemic invasion, and disease caused by these fungal pathogens. Buerth et al. [10] reported the role of *C. jadinii* and also *W. farinosa* as antagonistic agents against *C. albicans* by inhibiting its growth and morphogenesis, proposing their exploitation for the formulation of new prebiotic compounds and strategies to tackle candidiasis. *C. jadinii* was also described to secrete heterologous proteins into the growth medium, including lipase B from *Candida antarctica* (CalB) [56]. The signal sequence of the enzyme invertase, one of the most predominant proteins of the *C. jadinii* secretome, allowed high secretion levels of recombinant CalB [5]. Lipases have a great potential application in substitution therapies, where metabolic deficiency is overcome by external administration of these enzymes in diseased conditions [78]. Furthermore, lipase activity can go from activation of tumor necrosis factor, having a relevant role over the treatment of malignant tumors, to the treatment of gastrointestinal disturbances, digestive allergies, or dyspepsias [78]. In addition, CalB is involved in enzymatic resolutions, desymmetrization, and aminolysis events with application in not only pharmaceutical but also biotech industry, having a role in polymer production [78,79]. *C. jadinii* is also able to synthesize (R)-phenylacetylcarbinol (L-PAC), the pharmaceutical precursor for L-ephedrine and pseudoephedrine, relevant compounds used in the treatment of nasal congestion [80,81,82,83]. The function of 30 ATP-binding cassette transporters (ABC) transporters was studied by the amplification of predicted *C. jadinii* gDNA ORFs. The function of putative multidrug efflux pumps was evaluated by heterologous expression in *S. cerevisiae* ADΔ, a strain disrupted in seven of its major multidrug efflux pumps: Pdr5p, Pdr10p, Pdr15p, Snq2p, Pdr11p, Ycf1p, and Yor1p [84]. This strategy uncovered the mechanism of action of CjCdr1, *C. jadinii*’s closest homolog of the multidrug efflux pump *C. albicans* Cdr1. The characterization of *C. jadinii* multidrug efflux systems can turn *C. jadinii* into an appealing host for the development of novel antimicrobial agents [85], as it is imperative to understand the structure, function, and expression of multidrug efflux pumps in order to develop optimal novel antimicrobial agents.

### 6.2. Bioproduction of Valuable Compounds Using Cost-Effective Carbon Sources

Recombinant *C. jadinii* strains were developed for the production of a variety of compounds from food supplements such as vitamins (biotin) [86], carotenoids (lycopene, β-carotene, and astaxanthin) [60,68], to proteins (α-amylase, monellin) [64], antioxidant glutathione [87], polysaccharides (glucomannan) [88,89], organic acids (L-lactic acid) [44,59], and ribonucleic acids [5,24]. Additionally, the production of secreted enzymes such as invertase and phospholipase B (NBRC 1086 strain) [27,28] was also explored. Cells of *C. jadinii* DSM 2361 were successfully engineered for the secretion of *Penicillium simplicissimum* xylanase (PsXynA) to the culture medium [65] allowing cells to grow on xylan as the sole carbon source. Cells expressing the xylose reductase from *Candida shehatae* and the native xylitol dehydrogenase, in combination with further multiple site-directed mutations in coenzyme binding sites, resulted in the highest titer of 17.4 g/L of ethanol from 50 g/L of xylose in 20 h [90]. Organic acids present in industrial waste streams (e.g., acetic acid, propionic, or butyric acid) have been demonstrated to be suitable substrates for biomass production, reaching biomass yields varying from 30% to 40% in batch cultures, while in continuous cultures, an average of 44–55% was achieved [91,92]. Despite its already important role as an industrial microorganism, further developments are still necessary to fully explore the biotechnological potential of this yeast.

### 6.3. Industrial Applications—A Patent-View

In recent years, the applications of *C. jadinii* were extended to cosmetic and health care products and to the chemical-process industry for the production of chiral chemicals, as well as for agriculture and wine making. In this last application, *C. jadinii* yeast was used in the production of loquat wine, being introduced after *S. cerevisiae* fermentation to reduce acid content and enhance the aroma [93,94]. It is also used in another fermentation process, for the production of an alcohol-free fruit wine rich in lovastatin [95]. In the cosmetic industry, a β-D-glucan polysaccharide produced by *C. jadinii* was applied in formulations of several products, i.e., body lotions [96], sunscreen cream [97], facial cleanser [98], toner [99], eye cream [100], shampoo [101], body wash [102], and hand-care cream [103]. This bioproduct is mainly added for its properties as a moisturizing agent and for conferring oxidation and radiation resistance. *C. jadinii* was also used for the efficient production of a recombinant uricase, active in humans and with greater stability and/or activity than naturally occurring enzymes [104]. This enzyme can be used for the treatment of hyperuricemia-related diseases or other human pathological symptoms. Considering the chemical industry, the bioproduction of methyl fluorophenyl methyl propionate was achieved with a developed reduction method using *C. jadinii* as a biocatalyst [105]. The obtained chemical, (2S,3S)-3-(4-fluorophenyl)-3-hydroxy-2-methyl methyl propionate, is reported to be produced in high yield and with a high level of the enantiomeric excess rate. The wide applicability of this chiral building-block chemical can go from the synthesis of chiral drugs, fine chemicals to pesticides [105]. In agriculture, a consortium of strains that include *C. jadinii* was incorporated in an organomineral granular fertilizer containing among other components fulvic acid, and a natural mineral component (activated natural siliceous zeolite-containing rock). Its properties allow the reduction in the amount of fertilizer introduced in the soil with a prolonging action improved [106]. As *C. jadinii* is capable of efficiently converting a cadmium form from contaminated soil, it is now being proposed for soil bioremediation [107]. In addition, a microbial soil conditioner for lithified soil was also developed involving a *C. jadinii* strain along with *Bacillus megaterium*, *Bacillus subtilis*, *Rhodopseudomonas palustris*, and *Azotobacter chroococcum* species. The full interaction among the aforementioned strains was claimed to improve the soil ecological environment and alter the soil lithiation event, thereby contributing for the purpose of turning the soil suitable for farming [108].

## 7. Membrane Transporters Characterized in *C. jadinii*

**Figure 3:**
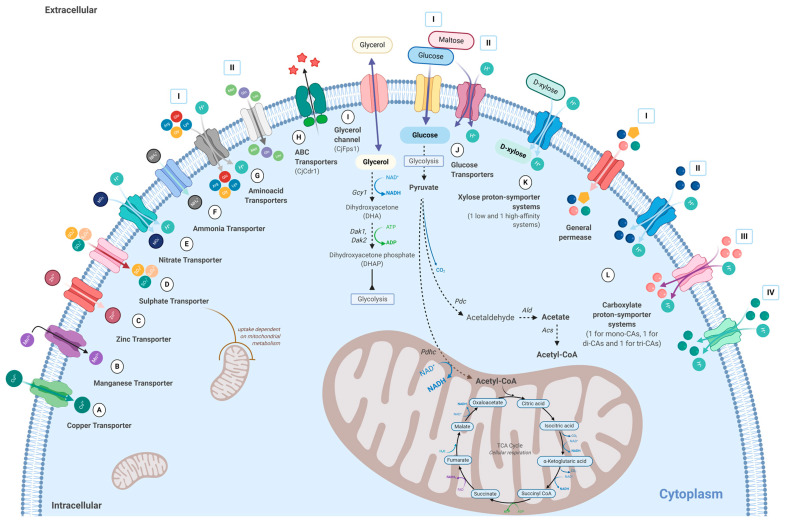
Plasma membrane transporters functionally characterized in *Cyberlindnera jadinii* (A–L). The main metabolic pathways of yeast general metabolism are also presented. Symbols represent the specificities uncovered for each of the protein-system: drugs—pink star, amino acids—yellow pentagon, monocarboxylic acids—blue filled circle, dicarboxylic acids—rose filled circle, and tricarboxylic acids—green filled circle. I-IV corresponds to different transporter systems with the same type of substrate G-I: amino acid proton symporter; G-II: facilitated diffusion of L-methionine, L-glutamine, and L-leucine; J-I glucose proton-symporter; J-II glucose facilitated diffusion; L-I facilitated diffusion of the undissociated form of carboxylic acids (general permease); L-II monocarboxylate proton symporter; L-III dicarboxylate-proton symporter; L-IV tricarboxylate-proton symporter. Initials stand for Mg^2+^, magnesium; Mn^2+^, manganese; SO_4_^2−^, sulfate; SO_3_^2−^, sulfite; S_2_O_3_^2−^, thiosulfate; NH_4_^+^, ammonia; NO_3_^−^, nitrate; Cu^2+^, copper; Zn^2+^, zinc; H^+^, proton; Gln, L-glutamine; Met, L-methionine; Leu, L-leucine; Arg, arginine; Lys, lysine; Gly, glycine; Glu, glutamate; *Gcy1*, glycerol dehydrogenase; *Dak1*, *Dak2* dihydroxyacetone kinases; *Pdhc*, pyruvate dehydrogenase complex; *Pdc*, pyruvate decarboxylase; *Ald*, aldolase; *Acs*, acetyl-CoA synthetase; *Acetyl-CoA*, acetyl coenzyme A; TCA cycle, tricarboxylic acid cycle; *NAD^+^*, reduced form from nicotinamide adenine dinucleotide; *NADH*, nicotinamide adenine dinucleotide; *FADH*, flavin adenine dinucleotide; *FAD^+^*, reduced form from flavin adenine dinucleotide; *ATP*, adenosine triphosphate; *ADP*, adenosine diphosphate; CO_2_, carbon dioxide; H_2_O, water.

The production of biocompounds in high yields requires the optimization of several processes, including membrane transport of solutes to improve the entrance of substrates in the cell, exchange of products within cell organelles, and the efflux of metabolites to the extracellular medium, increasing the cell’s tolerance to toxic final products, and decreasing downstream processing costs [109]. In *C. jadinii*, several plasma transporters were physiologically or genetically characterized (Figure 3).

In this yeast, copper (Cu^2+^) transport is biphasic, energy-dependent, and relatively specific. Uptake is inhibited completely by 2,4-dinitrophenol (DNP), but carbonyl cyanide m-chlorophenylhydrazone (CCCP) had relatively little effect (Figure 3A) [33,110]. The uptake follows a Michaelis-Menten kinetic with mean values for *K*_t_ = 3.1 µM and *V*_max_, 0.5 nmol min^−1^ per mg (dry wt.) and has an optimal pH between 5 and 5.5. No exchange of K^+^ for Cu^2+^ could be detected during Cu^2+^ uptake, and Cu^2+^ efflux from preloaded cells was not observed [33].

A high-affinity energy- and pH-dependent manganese (Mn^2+^) importer was reported in *C. jadinii* (Figure 3B) [111]. With an apparent half-saturation constant *K*_t_ of 16.4 nM and a *V*_max_ of 1.01 nmol min^−1^ mg^−1^ dry wt., this transporter was shown to be highly specific for Mn^2+^ uptake. Efflux studies demonstrated that the metabolic exchange of labeled ^54^Mn occurred to a small extent, being unaffected by a 100-fold molar excess of Mg^2+^, Zn^2+^, Ca^2+^, Co^2+^, Ni^2+^, and Cu^2+^, but inhibited 30–40% by a 1000-fold molar excess of Mg^2+^, Zn^2+^, Ca^2+^, Co^2+^, Ni^2+^ [110,111].

The zinc uptake-system described in *C. jadinii* is energy-dependent and apparently unidirectional as no exchange occurs between intracellular accumulated ^65^Zn and cold external Zn^2+^ (Figure 3C) [112]. This transporter exhibits a high-affinity for Zn^2+^ (*K*_m_ = 0.36 μM) with a *V*_max_ of 2.2 nmol min^−1^ per mg dry wt. of cells. The regulation of zinc homeostasis occurs either by altering the levels of a cytoplasmic zinc-sequestering macromolecule or by inhibition of zinc efflux through a membrane carrier [112,113,114].

The saturable and unidirectional sulfate transporter (Figure 3D) is pH-, temperature-, and energy-dependent with a *K*_m_ of 1.43 mM, being competitively inhibited by molybdate, selenate, thiosulfate, chromate, and sulfite [115,116]. The activity of this sulfate transporter is controlled by the pool of external sulfur compounds as well as by the mitochondrial metabolism [116].

The proton-symporter for nitrate (Figure 3E) is repressed by ammonium [117,118,119]. Ali and Hipkin [118] reported that the addition of 1, 2, or 10 mM ammonium resulted in an inhibition of nitrate uptake close to 30%. Studies using 3,3′-dipropylthiadicarbocyanine, a fluorogenic probe used to detect and measure alterations in transmembrane potential, indicated that the proton-linked uptake of nitrate, amino acids, or glucose during energy metabolism tended to depolarize the plasma membrane of *C. jadinii* cells [117].

An ammonia carrier (Figure 3F), revealed by spectrophotometry, presents a *K*_m_ of 1.0 µM of NH_4_^+^ [120]. Several amino acid proton-symporters (Figure 3G, I and II), namely, for arginine, lysine, glycine, and glutamate were uncovered in *C. jadinii* [121,122]. A permease for L-glutamine (*K*_m_ = 410 µM), a high (*K*_m_ = 23 µM) and low-affinity (*K*_m_ = 495 µM) transporter for L-methionine and a high (*K*_m_ = 5.6 µM) and a low affinity (*K*_m_ = 530 µM) for L-leucine were also described [123].

Thirty ABC transporters (Figure 3H) from different transporter subfamilies were found by homology search on the genome of the strain NBRC0988 [85]. The expression of the *C. albicans* Pdr1 homolog CjCdr1 in the *S. cerevisiae* ADΔ strain conferred resistance to geneticin (75 μg), micafungin (40 μg), and nystatin (500 μg). These two proteins present similar substrate specificity, although CjCdr1 is more resistant to Rhodamine 6G [85]. The *C. jadinii* aquaglyceroporin CjFps1 (Figure 3I) shares 38% of identity with the *S. cerevisiae* Fps1. The heterologous expression of *CjFPS1* in a glycerol-consuming *S. cerevisiae* wild-type strain (CBS 6412-13A) promoted cellular growth improvement on glycerol as the sole carbon source [124].

Two different systems were found for glucose transport in *C. jadinii*, a proton-symporter and a facilitated diffusion (Figure 3J) [125,126,127], however, the respective genes remain unidentified. The high-affinity proton-symporter presents a *K*_m_ of 15 µM glucose, displays a stoichiometry of 1:1, and is partially constitutive, appearing in cells grown on gluconeogenic substrates such as lactate, ethanol, and glycerol. This transporter is repressed by high glucose concentration but is induced by glucose up to 0.7 mM [125], a behavior also found for the maltose-uptake system, indicating that both systems share a common glucose control pathway [125,128]. Barnett and Sims (1976) proposed that the differences in glucose vs. maltose affinity can be due to allosteric mechanisms associated with a multimeric transporter or to the hysteretic behavior of a monomeric transporter [126], conditions that have not yet been solved as the correspondent genes(s) remain unidentified. The facilitated diffusion mechanism (Figure 3J-II) is found in cells growing on glucose at concentrations higher than 10 mM and presents complex kinetics of glucose transport whose *K*_m_ oscillates between 2 and 70 mM [125,128]. It was also reported that uric acid enters *C. jadinii* cells by the glucose-dependent active transport [129].

The low- and the high-affinity systems for D-xylose transport (Figure 3K) with *K*_m_ values of 67.6 ± 3.2 and 1.9 ± 1.2 mM, respectively, act as proton-symporters with distinct modes of regulation. The starvation of glucose-grown cells decreases the *K*_m_ value of the low-affinity system (*K*_m_ = 10.5 ± 2.6 mM) [130]. The high-affinity system found during starvation requires protein synthesis and is inactive when cells are exposed to glucose, through a process independent of protein synthesis. Glucose and acetic acid inhibited both the high- and low-affinity xylose transport systems [130].

Four mediated transport systems for organic acids are described in *C. jadinii* (Figure 3L) [29,30,31]. The monocarboxylate proton symport, shared by the L- and D-lactate (*K*_m_ 0.06 mM), pyruvate (*K*_m_ 0.03 mM), propionate (*K*_m_ 0.05 mM), and acetate (*K*_m_ 0.1 mM) (Figure 3L-II) is active over a pH range of 3.0–6.0, with an optimum of activity at pH 5.0 [29]. The dicarboxylate-proton symporter (Figure 3L-III) is shared by L-malate (*K*_m_ 4.0 ± 0.5 µM), succinate (*K*_m_ 0.03 ± 0.01 mM), fumarate, oxaloacetate, and α-ketoglutarate [30,31,131]. The tricarboxylate-proton symporter (Figure 3L-IV) presents a *K*_m_ of 0.056 mM for citrate and is competitively inhibited by isocitric acid, while aconitic, tricarballylic, trimesic, and hemimellitic acids did not affect citrate uptake [30]. All these carboxylic acid transporters are inducible by the respective substrates, being subjected to glucose repression as well as by acid concentrations higher than 3% (w/v) [31]. The facilitated diffusion for the undissociated form of the acids (Figure 3L-I), which is likely to operate as a general organic permease, is active at pH below 5.0. It accepts mono-, di-, and tricarboxylates as well as glycine and glutamic acid [31,132]. The following kinetic parameters were obtained at pH 3.0: (a) *V*_max_ 0.516 nmol of malic acid s^−1^ per mg (dry wt. of cells) and *K*_m_ 1.529 ± 0.024 mM malic acid, (b) *V*_max_ 0.585 nmol of succinic acid s^−1^ per mg (dry wt. of cells) and *K*_m_ 1.789 ± 0.089 succinic acid, and (c) *V*_max_ of 1.14 nmol of s^−1^ per mg (dry wt. of cells) and *K*_m_ of 0.59 mM citric acid [29,30,31]. Despite being functionally characterized for several years, the genes encoding these proteins remain to be identified [133]. Recently, our group identified six genes homologous to Sc*ATO1* and six genes homologous to Sc*JEN1* (unpublished results). In *S. cerevisiae*, these two genes encode distinct monocarboxylate transporters [134,135] and its homologs, present in bacteria, archaea and eukaryotes, are able to transport mono- and dicarboxylates [136,137,138,139,140,141,142]. The expression of membrane transporters allied to metabolic engineering tools is crucial to develop new and more efficient strains to produce bio-based compounds. Thus, increased knowledge of transporter proteins will enable the development of improved cell factories [109,143,144].

## 8. Conclusions and Future Perspectives

As biotechnological applications expand, it becomes necessary to explore novel expression hosts as more efficient and robust microbial cell factories are demanded [123,124,125]. Over the years, *Cyberlindnera jadinii* has been widely explored as a source of single-cell protein, having the ability to produce vitamins (e.g., biotin), organic acids (e.g., glutamate), and proteins (e.g., enzymes). The capacity to utilize and degrade a great variety of carbon sources and its natural ability to produce significant compounds make *C. jadinii* an attractive microorganism for industrial applications [5,7,8,10]. Additionally, several features turn this yeast an ideal platform for biotechnological processes, like the higher level of tolerance to changes occurring during growth and multiplication conditions [5,55]. However, *C. jadinii* is still lagging behind when compared to other non-*Saccharomyces* yeasts, mainly due to the inexistence of extensive knowledge on its metabolism, regulatory networks, and transport mechanisms. The genomic characterization of several strains is also necessary to reveal the genetic features underlying the existing interspecies variability, particularly between its teleomorph and anamorph state. The potential of this yeast as a therapeutic agent is due to its already known antagonistic effects on human pathogens and utilization as a probiotic agent. Its protein secretion system is another attractive feature for the heterologous expression of soluble proteins. Nonetheless, only with the improvement of advanced genetic manipulation systems and the development of synthetic biology tools will the full exploitation of *C. jadinii* biotechnological potential be achieved. These and other advances will certainly allow *C. jadinii* to become a robust microbial cell factory in an expanding era of metabolite bioproduction.

## Figures and Tables

**Table 1:** Main *C. jadinii* (former *C. utilis*) strains described in literature.

Nomenclature in Literature	Current Nomenclature	Isolation Source	Reference
*Candida utilis* NBRC 0988	*C. jadinii* ATCC 9950; CBS 5609; DSM 2361; NBRC 0988; NCYC 707; NRRL Y-900	Yeast factory in Germany	[24]
*C. utilis* ATCC 9256 ^a^	*C. jadinii* NRRL Y­1084; CBS 841; CCRC 20334; DSM 70167; NCYC 359; VKM Y­768; VTT C­79091	Unknown	[25,26]
*C. utilis* ATCC 9226 ^a^ NBRC 1086	*C. jadinii* VTT C-71015; FMJ 4026; NBRC 1086	Unknown	[25,27,28]
*C. utilis* IGC 3092	*C. jadinii* PYCC 3092; CBS 890; VKM Y-33	Unknown	[29,30,31]
*C. utilis* CCY 39-38-18	*C. jadinii* CCY 029-38-18 ^b^	Unknown	[32]
*C. utilis* NCYC 708	*C. jadinii* NCYC 708; ATCC 42181; CBS 5947; VTT C-84157	Unknown	[33]
*C. utilis* CBS 4885 NRRL Y-6756	*C. jadinii* CBS 4885; NRRL Y-6756; NBRC 10708	Cow with mastitis	[34]
*C. utilis* CBS 567 NRRL Y-1509	*C. jadinii* CBS 567; NRRL Y-1509	Yeast deposit in distillery	[34]
*C. utilis* CBS 2160	*C. jadinii* CBS 2160	Flower of *Taraxacum* sp.	[34]
*C. utilis* CBS 621	*C. jadinii* CBS 621; NRRL Y-7586; ATCC 22023; PYCC 4182	Yeast factories	[35]
*C. utilis* CBS 1600	*C. jadinii* CBS 1600; NRRL Y-1542; ATCC 18201	Pus of a woman abscess	[16]

^a^ This strain has been discontinued in ATCC. ^b^ The strain number reported in the literature is not available in the Culture Collection of Yeasts (CCY), all *C. jadinii* strains are registered as 029-38-XX, including *C. jadinii* 029-38-18, the likely match to CCY 39-38-18.

**Table 2:** Genomic features of three *C. jadinii* strains and the reference *S. cerevisiae* strain S288c.

Components	*Cyberlindnera jadinii* Strains			*Saccharomyces cerevisiae* S288c
	NBRC 0988	CBS 1600	NRRL Y-1542	
**NCBI assembly reference**	GCA_000328385.1	GCA_001245095.1	GCA_001661405.1	GCA_000146045.2
**Assembly level**	Chromosomes	Scaffold	Scaffold	Complete genome
**Genome size**	14.3 Mb	12.7 Mb	13.0 Mb	12.2 Mb
**Genes ^a^**	8864	5566	6184	6002
**No of scaffolds ^b^**	1002	7	76	17
**Scaffold N50 ^b^**	189,765	2,123,196	700,888	924,431
**No. of contigs ^b^**	1163	91	392	17
**Contig N50 (bp) ^b^**	158,681	287,918	111,555	924,431
**No. of chromosomes**	13	–	–	16
**GC-content (%)**	44.7	44.5	44.6	38.3
**Total of CDS ^a^**	8646	5057	6032	5771
**Gene annotation**	[24]	[16]	[37]	[47,48]

^a^ Total number of predicted genes and protein-coding genes (CDS) are taken from original publications or subsequent annotations. ^b^ Data retrieved by Joint Genome Institute (JGI)—Integrated Microbial Genomes & Microbiomes system (https://img.jgi.doe.gov/).
